# Weak global trade‐off between frost and drought resistance in trees

**DOI:** 10.1111/nph.70718

**Published:** 2025-11-09

**Authors:** Maximilian Larter, Guillaume Charrier, Sylvain Delzon, William Hammond, Anne Baranger, Constance Bertrand, Nicolas Martin‐StPaul, Georges Kunstler

**Affiliations:** ^1^ Univ. Bordeaux, INRAE, BIOGECO 33600 Pessac France; ^2^ Univ. Clermont Auvergne, INRAE, PIAF 63000 Clermont‐Ferrand France; ^3^ University of Florida Gainesville FL 32608 USA; ^4^ Univ. Grenoble Alpes, INRAE, LESSEM 38400 St‐Martin‐d'Hères France; ^5^ Univ. Rennes, CNRS, ECOBIO 35700 Rennes France; ^6^ URFM, INRAE 84914 Avignon France

**Keywords:** drought stress, embolism resistance, frost resistance, functional traits, trade‐off, trees, xylem

## Abstract

- Drought and frost stresses play important roles in determining species distributions, especially at range margins. Understanding how stress resistance traits interact to determine vulnerability to climate change is critical.
- We developed a large global database of published and new measurements of drought resistance (xylem embolism resistance; P_50_) and frost resistance (electrolyte leakage; LT_50_), and investigated evolutionary trade‐offs using Bayesian phylogenetic quantile regressions.
- Across all woody biomes, P_50_ ranged from −1 to −19 MPa, and LT_50_ from 0 to below −80°C with conifers generally more resistant than angiosperms. We found a weak trade‐off between drought and frost resistance: Drought‐resistant species tend to be less frost hardy, and vice versa. There are few species resistant to both stresses (e.g. junipers). Including the phylogeny reduced the strength of the relationship, reflecting the phylogenetic signal for these traits. We did not find any strong effects of LT_50_ on growth‐related traits, but drought resistance is associated with denser wood, smaller conduits, shorter stature and lower specific leaf area.
- While we show a trade‐off between frost and drought resistance, our study does not support the global fast–slow economics spectrum. Our results have implications for forests experiencing hotter, drier summers and potentially damaging late frosts.

## Introduction

Climate extremes such as freezing temperatures and severe heatwaves and droughts play a leading role in determining tree species distributions, which are mainly limited at the cold margins by frost resistance and on the warm margins by drought resistance (Richardson & Bond, 1991; Hampe & Petit, 2005; Choat *et al*., 2008; Körner, 2021; Baranger *et al*., 2024). Climate change has already deeply modified rainfall and temperature patterns in many areas of the world, and increased the stochasticity and duration of extreme climate events such as droughts and heatwaves (Masson‐Delmotte *et al*., 2021; King *et al*., 2024). It is causing large‐scale tree mortality events in all forest ecosystems following hot droughts, with prolonged low rainfall compounded by higher temperatures (Allen *et al*., 2010; Anderegg *et al*., 2015; Senf *et al*., 2020; Hammond *et al*., 2022; Hartmann *et al*., 2022). A general warming pattern could also increase vulnerability to sudden autumn and winter cold snaps and spring frosts (Matusick *et al*., 2014; Vanoni *et al*., 2016; Greco *et al*., 2018; Körner, 2021; Vitasse *et al*., 2025). From boreal to Mediterranean ecosystems, forests are therefore likely to be exposed to more frequent, alternating frost and drought stresses, with compounding effects on productivity and survival (Vitasse *et al*., 2019; Charrier *et al*., 2021), and their distributions. By their sessile nature, woody perennial plants must survive seasonal frost and drought, and compete for resources with other species in order to grow and reproduce. This leads to balancing selection pressures: between different stress resistance traits (e.g. frost vs drought resistance) on the one hand; and between stress resistance and growth or reproduction traits on the other hand (Grime, 1977; Reich, 2014). In particular, direct and indirect fitness costs of functional traits related to frost and/or drought resistance can result in trade‐offs within the plant, in turn determining their ability to compete and survive, especially at their distribution margins (Sanchez‐Martinez *et al*., 2023; Baranger *et al*., 2024). Understanding how tree functional traits interact to determine species‐specific response to extreme frost and drought is crucial in order to be able to predict climate change impact on forest dynamics at local and continental scales (Pollock *et al*., 2012; Stahl *et al*., 2014; Aubin *et al*., 2016; Wiens, 2016).

In woody plants, hydraulic failure due to xylem embolism has emerged as a ubiquitous mechanism in drought‐induced mortality (Adams *et al*., 2017; McDowell *et al*., 2022). During droughts, heat‐driven water use and soil water depletion drive an increase in xylem tension in the sap stream, in turn causing air bubbles to be pulled into functional conduits through pits – the pores connecting xylem conduits (Sperry & Tyree, 1988; Tyree & Sperry, 1989; Delzon *et al*., 2010). Xylem embolism resistance has been identified as a key functional trait directly linked to mortality (Brodribb & Cochard, 2009; Urli *et al*., 2013; Anderegg *et al*., 2016; Charrier *et al*., 2018) and broadly correlated with aridity (Brodribb & Hill, 1999; Maherali *et al*., 2004; Delzon *et al*., 2010; Larter *et al*., 2017; Skelton *et al*., 2021). Although additional parameters are needed to determine the time to death during drought (Martin‐StPaul *et al*., 2017; Cochard *et al*., 2021; McDowell *et al*., 2022; Mantova *et al*., 2023; Petek‐Petrik *et al*., 2023), and other strategies regarding extreme drought exist (e.g. avoidance), P_50_, that is the xylem pressure inducing a 50% drop in hydraulic conductance, is the most widely measured, highest‐confidence estimate of xylem resistance to drought in trees. Negative temperatures can damage plants through: the formation of intracellular ice, lethally disrupting cell membranes, and extracellular ice, inducing intensive tissue dehydration, which can also be lethal to plant cells (Sakai & Larcher, 1987a); and winter (or freeze–thaw) xylem embolism when dissolved gases come out of solution upon freezing, in turn nucleating cavitation and expanding to embolize conduits upon thawing (Sperry & Sullivan, 1992; Davis *et al*., 1999; Hacke & Sperry, 2001; Pittermann & Sperry, 2003; Charrier *et al*., 2013a, 2014). At the onset of winter, environmental cues trigger cold acclimation (hardening), by lowering the freezing point of the intracellular compartment, mobilizing internal carbon resources and decreasing bulk water content, synthesizing antifreeze proteins and changing the composition of cell membranes to maintain their fluidity (Weiser, 1970; Sakai & Larcher, 1987b; Baffoin *et al*., 2021; Charrier *et al*., 2021). These processes allow plants to tolerate the formation of extracellular ice crystals, and living cells of hardened tissues to survive negative temperatures over winter – in some species down to the temperature of liquid nitrogen (Sakai & Larcher, 1987a). Maximum frost resistance is measured by quantifying damage to winter‐hardened tissues after exposure to extreme negative temperatures (Dexter *et al*., 1932; Aniśko & Lindstrom, 1995; Kovaleski & Grossman, 2021). By contrast, susceptibility to winter embolism depends on plant water status and the number of freeze–thaw cycles (Sperry & Sullivan, 1992; Sperry *et al*., 1994), and comparing species resistance to freeze–thaw embolism and relating it to species ecology is challenging. Xylem conduit diameter is broadly correlated with the minimum annual temperature of the species' distribution (Zanne *et al*., 2018), and we can expect a correlation between resistance to freeze–thaw embolism with narrow xylem conduits and frost resistance of living tissues, since species adapted to extreme frost also experience multiple freeze–thaw cycles every winter (McCulloh *et al*., 2023). Trees' ability to avoid damaging xylem embolism during drought and to minimize cell death during extreme cold events are both recognized as critical traits for survival. Yet, these traits have rarely been examined together in a comparative framework (Charrier *et al*., 2021; McCulloh *et al*., 2023), limiting our understanding of how drought and frost resistance traits evolve in stress tolerance syndromes (Puglielli *et al*., 2021; Volaire *et al*., 2023; Pavanetto *et al*., 2024).

From a mechanistic point of view, we can expect synergies between drought‐ and frost resistances in woody plants across different compartments and processes. Both stresses trigger shared signaling pathways, induce similar regulatory and molecular responses, impact xylem water transport and induce tissue dehydration (Beck *et al*., 2007; Körner, 2016; Zhang *et al*., 2020; Charrier *et al*., 2021), ultimately leading to cell death (Sakai & Larcher, 1987a; Mantova *et al*., 2023). At the vascular level, both reduce xylem water flow via drought‐ and freeze–thaw‐induced embolism (Charrier *et al*., 2021). This leads to potential indirect coordination between frost and drought stress resistance, as strategies for resisting summer embolism could make winter embolism less likely – and vice versa (Christensen‐Dalsgaard & Tyree, 2013; Torres‐Ruiz *et al*., 2016). Drought‐resistant species tend to have xylem conduits with thicker cell walls relative to lumen size to withstand very negative xylem pressures (Pittermann *et al*., 2006; Sperry *et al*., 2006; Bouche *et al*., 2014; Larter *et al*., 2017). Because of the scaling of pits and pit membranes with conduit dimensions, we expect smaller conduits (with thicker walls) to favor both resistance to drought and freeze–thaw embolism (Tyree & Cochard, 1996; Mayr *et al*., 2006; Zhang *et al*., 2024; Pan *et al*., 2025). Woody tissue of drought‐resistant species can have a higher parenchyma fraction (Morris *et al*., 2016; McDowell *et al*., 2022), aiding both hydraulic capacitance and nonstructural carbohydrate (NSC) storage. This interdependency between water and carbon economies likely contributes to survival during drought or freezing weather (Bond & Midgley, 2001; McDowell *et al*., 2022; Trueba *et al*., 2024). A shared, albeit limited pool of NSCs necessary for drought and cold stress responses could facilitate surviving hot dry summers and harsh winters (Blumstein *et al*., 2023). Mild summer drought can actually cause an increase in carbohydrate reserves, because growth stops before photosynthesis (McDowell, 2011; McDowell *et al*., 2011; Muller *et al*., 2011; Hartmann *et al*., 2018), and drought stress can induce an increase in frost hardiness in some species, with reduced water content and higher solute concentration lowering the freezing point (Charrier *et al*., 2021). From an evolutionary perspective, woody angiosperms likely adapted to freezing environments by co‐opting adaptations to seasonal drought, such as deciduousness or narrow embolism‐resistant conduits (Preston & Sandve, 2013; Zanne *et al*., 2014). Conifer physiology and anatomy allow them to tolerate cold, dry, nutrient‐poor environments, maintaining dominance in these habitats during the radiation of angiosperms (Bond, 1989; Augusto *et al*., 2014). Plant species with ‘slow’ ecological strategies are often thought of as slow‐growing, long‐lived and stress‐resistant species (Volaire *et al*., 2023). In this framework, investment in resistance traits (either frost or drought resistance, or both) could limit energy available for growth, resource acquisition and reproduction, which is sometimes called a physiological or allocation trade‐off (Grime, 1977; Stearns, 1992; Willi & Van Buskirk, 2022). Reflecting ecological strategies along the leaf and wood economics spectra (Wright *et al*., 2004; Chave *et al*., 2009), an evolutionary correlation could emerge between drought resistance and frost resistance, mediated by cost effects on growth traits such as specific leaf area, maximum plant height, wood density, xylem conduit diameter and hydraulic conductivity (Carlquist, 1975; Poorter *et al*., 2008, 2009; Martínez‐Vilalta *et al*., 2010; Gleason *et al*., 2016; Hietz *et al*., 2017; Simovic *et al*., 2024).

However, a direct physiological trade‐off between drought and frost resistance may arise due to costs associated with establishing resistance to one type of stress‐limiting resources available for resisting the other, as well as for optimizing other fitness‐related functional traits (Stahl *et al*., 2014). Species in dry environments invest significantly in xylem cell wall thickness, and dense wood with a high proportion of fibers (due to mechanical stress during drought). This high demand for structural carbon and a reduced carbon storage compartment reduce the availability of NSCs for the energy‐intensive cellular and molecular changes that occur during early‐winter hardening (Sakai & Yoshida, 1968; Sakai & Larcher, 1987c; Charrier & Améglio, 2011; Charrier *et al*., 2013b, 2021; Baffoin *et al*., 2021). Prolonged summer drought can deplete NSC reserves when respiration outpaces photosynthesis, impacting winter frost response (McDowell *et al*., 2011, 2022; Harbol *et al*., 2023). Similarly, periods of cold weather also consume NSCs, decreasing subsequent growth and summer resilience to drought (D'Andrea *et al*., 2020, 2021; Camarero *et al*., 2023; Alderotti *et al*., 2024). This hypothetical functional trade‐off between drought resistance and frost resistance precludes the existence of ‘poly‐tolerance’ phenotypes (McCulloh *et al*., 2023), and across species, we may then find a negative relationship between drought resistance and frost resistance.

Finally, several lines of evidence support a more nuanced picture. For example, exposure to drought has been shown to promote frost resistance in some species (Medeiros & Pockman, 2011; Sierra‐Almeida *et al*., 2016; Sumner *et al*., 2022), but not all (Fernández‐Pérez *et al*., 2018). Comparative studies have shown that drought and frost resistance traits are sometimes correlated across species (Charrier *et al*., 2014; Pescador *et al*., 2016; Visakorpi *et al*., 2024), but not always (Charra‐Vaskou *et al*., 2012; Rueda *et al*., 2017; di Francescantonio *et al*., 2020). The relationships between different abiotic stressors also appear complex, with a drought–frost resistance trade‐off modulated by interaction effects with other stresses such as waterlogging or shade (Laanisto & Niinemets, 2015; Puglielli *et al*., 2021). This complexity could arise because the selective pressures induced by drought and frost mostly do not overlap temporally (i.e. summer vs winter) and spatially – for example at opposite ends of the species ranges. Therefore, if resistance traits are not tightly linked, for example through pleiotropy, we could expect all trait combinations to exist.

In this study, we constructed a new global database of resistance to drought‐induced xylem embolism and frost resistance in woody plants, building on existing knowledge and adding a large number of new measurements. We applied strict quality control filters on published data to deal with known measurement artifacts and biases. We first tested the hypothesis that: (1) there is a strong direct trade‐off between drought resistance and frost resistance, with the absence of polystress resistance traits combinations (McCulloh *et al*., 2023). This would be driven by evolutionary constraints between functional traits, which can be restricted to the tails of trait distributions, that is toward climatic extremes, and not be apparent when using traditional means‐based regression methods (Cade & Noon, 2003). Specifically, embolism resistance could exert constraints on the evolution of frost resistance in dry environments, whereas frost resistance evolution could be driven by other constraints in more mesic environmental conditions (e.g. by nutrient availability or shade). Furthermore, the shared evolutionary history of related species can complicate species‐level analyses, either obscuring patterns or leading to spurious conclusions (Felsenstein, 1985; Losos, 2008, 2011; Revell & Harmon, 2022). To account for these factors, we used flexible Bayesian generalized mixed models, allowing us to include phylogenetic structure as a random effect, and examine relationships between variables at different quantiles of their distributions. Second, in line with a survival vs growth trade‐off, we further expected (2) a trade‐off between drought and frost stress resistance traits on the one hand and traits enhancing resource acquisition or competitive ability such as conduit diameter, hydraulic efficiency, wood density, maximum height or specific leaf area on the other hand.

## Materials and Methods

### Frost resistance database

We obtained frost resistance data from the literature, using search terms: (1) “frost”, “cold”; and (2) “resistance”, “tolerance”, or “hardiness”. We limited our search to trees, shrubs and woody species. Overall, we collected data from *c*. 200 references in tables, supplementary data or directly from figures when necessary using WebPlotDigitizer (Rohatgi, 2022). From these papers, combined with unpublished data (G. Charrier, pers. comm.), we accumulated a database of *c*. 4000 measurements in over 600 species. We also recovered as much metadata as possible regarding the experimental setup, including which technique was used, the date and location of the study, the age of the plant, which organ/tissue was used and other parameters of the experiment, such as the rate of temperature change and the duration of exposure at the lowest temperature.

As a first step, based on the date of the study, we removed all measurements that were not from the middle of winter so that natural hardening had occurred, and the late‐winter dehardening process had not started. These measurements included data from various plant organs (mainly leaves, stems and buds), and used different techniques. Briefly, two main methods exist in the literature that both examine samples for damage after exposure to cold temperatures. First, the visual scoring (VS) technique is based on the visual examination of multiple tissue samples exposed to different cold temperatures, and LT_0_ (°C) is recorded as the coldest temperature at which the samples did not show cell damage, usually a visible necrotic discoloration of the tissue 1 or 2 d after cold exposure (Sakai & Larcher, 1987a). The electrolyte leakage (EL) technique employs a similar cold temperature gradient, and quantifies the release of electrolytes from plant cells as they die following exposure to cold temperatures. From the sigmoid response curve of the relationship between the relative EL (REL) obtained and the minimum temperature of exposure, we can derive the inflection point called LT_50_ (°C), that is the temperature at which 50% of REL is reached (Supporting Information Methods S1). These two techniques result in critical temperature thresholds that describe the species' frost resistance. Although measured differently, LT_0_ and LT_50_ were well‐correlated across species considering measurements across all organs (*R* ^2^ = 0.34; *P* < 0.001; *n* = 105 species; Fig. S1), with LT_0_ at higher temperatures on average than LT_50_ for the same species and organ, allowing us to keep data from both methods in subsequent analyses.

Among branches, buds and leaves, we found LT_0_ and LT_50_ to be generally comparable (Fig. S2). However, to ensure as much homogeneity as possible while maximizing the number of species in the dataset, we proceeded as follows: Within a species, we averaged all data grouping by organ and by method, resulting in a maximum of six different values per species (branch/bud/leaf by LT_0_/LT_50_). We prioritized measurements using the EL technique, which had protocols that are more consistent across studies (freezing and thawing rates will be discussed later). Branch data were much more common in the database, and we avoided a bias toward conifers (with fewer deciduous species, therefore with more leaf frost resistance data than for angiosperms) by placing leaf data last. Thus, we selected which value to use based on availability in this order: 1: LT_50_ in branches (144 species in the final dataset), 2: LT_50_ in buds (17 sp.), 3: LT_0_ in branches (228 sp.), 4: LT_0_ in buds (34 sp.), 5: LT_50_ in leaves (55 sp.) and 6: LT_0_ in leaves (5 sp.).

Finally, we investigated the impact of two factors likely to influence frost resistance. We split data based on the age of the plants, selecting on the one hand data collected from adult or mature individuals, and on the other hand data from experiments on juvenile and/or potted plants, and data sources not reporting plant age. Additionally, slow freezing and/or thawing rates can artificially induce cold hardening, whereas high rates can artificially increase the probability of intracellular ice formation or a strong reflux of melted water before the membrane of the protoplasts regained its original area (Weiser, 1970; Sakai & Larcher, 1987b). We therefore also compared data with controlled, gradual rates of temperature change, with data with estimated rates below 0.2°C h^−1^ and over 9°C h^−1^. This resulted in a reduced filtered dataset of average frost resistance with fewer species, which we then compared with the full dataset. The overall differences across species in both datasets are minute, with a regression line between the two values close to the 1 : 1 line (regression slope = 0.926; *R* ^2^ = 0.68, *P* < 0.001, *n* = 39; Fig. S3). Therefore, to keep as much data as possible, we ran the subsequent analyses on the full dataset.

We also made new measurements for 79 species (Table S1; Methods S1), focusing on adding species from drier areas (temperate and Mediterranean climates), which were underrepresented in frost hardiness studies. We only used the EL technique on stems (Zhang & Willison, 1987), modified from the protocol described elsewhere (Baffoin *et al*., 2021), and added these measurements to those from the literature, resulting in a final database containing data for 483 species, from 74 families and 193 genera.

### Drought resistance database

We built a large database of embolism resistance, based on previous efforts (Choat *et al*., 2012; Hammond *et al*., 2021), combined with other unpublished sources. The Xylem Functional Traits (XFT) database (https://xylemfunctionaltraits.org) contains published data of hydraulic vulnerability to drought (Choat *et al*., 2012) and is in the process of being updated (Hammond *et al*., 2021). Since this database included a number of different measurement techniques, some of which can induce artifacts that overestimate vulnerability to embolism (Cochard *et al*., 2010; Wheeler *et al*., 2013; Torres‐Ruiz *et al*., 2015), we applied strict quality control filters. First, we used the database's curve shape variable to remove vulnerability curves that were not sigmoid in shape, which is symptomatic of the ‘open‐vessel’ artifact. We also filtered out many ‘r‐shaped’ curves that were not initially flagged in the database. From this database, we kept data for woody species, resulting in 2193 data points from 1035 species.

For species with data from different methods, we compared the data (Fig. S4) and applied selection criteria as follows: (1) We averaged stem P_50_ data from centrifuge‐based methods (in conifers and short‐vessel species), and in all species from noninvasive techniques such as the optical technique or microcomputed tomography (μCT), and when these were all unavailable; (2) we used mean stem P_50_ from the bench‐top dehydration technique; then (3) we used leaf P_50_ data; and finally, (4) we used stem data from the air‐injection techniques. When P_50_ obtained with these selection criteria was higher than Ψ_TLP_, we removed it from the database.

Finally to add as many species as possible and to increase coverage of long‐vesseled angiosperms, we compiled additional data from other sources. From the Caviplace laboratory (Phenobois Platform, Bordeaux, France https://phenobois.hub.inrae.fr/), we added unpublished stem P_50_ for 123 conifers (Larter, 2016). We added leaf and stem P_50_ data for 33 species from a recent review of noninvasive measurement techniques (Cardoso *et al*., 2022), as well as 51 species from a separate curated database (Martin StPaul, pers. comm.). We also added new measurements using the flow‐centrifuge technique (Burlett *et al*., 2022), focusing on conifers and species with short vessels for which we already had frost resistance data (Table S2; Methods S1).

Overall, with over 900 species from the curated XFT database, the embolism resistance dataset contained P_50_ values for 1145 species from 116 families and 450 genera.

### Other traits

To examine the trade‐off between species' ability to resist stresses and their ability to grow and compete with their neighbors, we obtained the following functional trait data using available published databases: Xylem mean conduit diameter (μm) and xylem‐specific conductivity (Ks; kg s^−1^ MPa^−1^ m^−1^) were taken from physiological studies in the XFT database (Hammond *et al*., 2021). We also added xylem conduit diameter data from a recent review of the links between xylem structure and function (Lens *et al*., 2022), and conifer maximum xylem‐specific hydraulic conductivity (Ks) from the CaviPlace laboratory database (Delzon *et al*., 2010; Larter, 2016).

For wood density (g cm^−3^), we used the Wood Economics Spectrum database (Chave *et al*., 2009), with additional data taken from the XFT and CaviPlace laboratory databases.

For plant maximum height (m), we obtained maximum height for 111 species from an allometry modeling approach (Touzot *et al*., 2025). This reference provides estimates of maximum height as the asymptotic height parameter of an allometric model fitted to individual tree measurements of stem diameter and height from forest inventory plots in North America and Europe. To complete the dataset, we then obtained height data from Tallo (Jucker *et al*., 2022) and TRY (Kattge *et al*., 2020). After removing outliers (with the ‘height_outlier’ variable), we removed all species with fewer than 100 data points. There is a large error associated with extreme size measurements, and these data contain many small values, which impact distribution‐based metrics such as percentiles (Qiu *et al*., 2022; Journé *et al*., 2024). We therefore used an order statistic, and computed estimates for maximum height for 611 and 210 species for Tallo and TRY data, respectively, by using the height of the 10th tallest individual of each species. These two different estimations (modeling and measured height data) of maximum height are reasonably well‐correlated, although the measured data are generally lower across species (*R* ^2^ = 0.30***; Fig. S5), with some outliers in gymnosperms, which are much taller according to the modeling approach. We checked these data against information for maximum size from the Gymnosperm Database (Earle, 2024) and found them much closer to the model maximum height than the values derived from field measured tree heights. For these reasons, we chose to use the modeling data when available, and the 10^th^ ranked‐highest measured value for the other species. Once matched to the resistance trait data, this resulted in data for 300 species, of which 70 were from the modeling approach (Touzot *et al*., 2025) and the rest from the measured height approach.

For specific leaf area (m^2^ kg^−1^), and leaf area (mm^2^), we used data extracted from TRY (Kattge *et al*., 2020), with additional data from the Botanical Information and Ecology Network (BIEN; https://bien.nceas.ucsb.edu/bien/) using the R package rBIEN (Maitner *et al*., 2018).

### Climate

For all species in the physiological traits databases, we obtained climate data across the species range. We downloaded occurrences from The Global Biodiversity Information Facility (GBIF, 2023), and ran error‐filtering methods available in the rgbif package (Chamberlain *et al*., 2023). For species with large numbers of records, we resampled the occurrences with random draws, keeping a maximum of 15 000 records per species, and keeping a minimum distance of 10 km between points (Aiello‐Lammens *et al*., 2015) to avoid oversampling of highly visited areas of species ranges. From these sets of point coordinates for each species, we extracted climate information from different sources. First, we computed the mean annual precipitation (MAP; mm yr^−1^) and mean annual temperature (°C) from the Worldclim extrapolated climate layers at a resolution of 2.5 min (Fick & Hijmans, 2017) for each species.

As a better measure of habitat aridity than MAP, we used the Aridity Index (AI; no unit) from the Global Aridity Index and Potential Evapotranspiration Climate Database v3 (Zomer & Trabucco, 2022). AI is based on MAP divided by mean annual potential evapotranspiration and quantifies the availability of precipitation relative to atmospheric water demand. For this climatic variable, we estimated the 5^th^ percentile of AI across each species distribution, to quantify the driest edge of the species climate envelope.

Similarly, to obtain an estimate of the absolute lowest temperature experienced by each species, we obtained daily minimum temperature records from the CHELSA dataset (Karger *et al*., 2023), using the global daily minimum near‐surface air temperature (‘tasmin’) variable for the period from 1980 to 2005. From this series of monthly data series over the period, we extracted the minimum daily temperature value to a raster layer using the raster package in R (Hijmans, 2025). We then matched this minimum temperature layer to each location from the GBIF occurrence dataset. Then, for each species, we computed the 5^th^ percentile of minimum daily temperature from the species distributions. These variables were then used to investigate the relationships between extreme climate and corresponding resistance traits (Fig. S6).

### Statistics

To test for an evolutionary trade‐off between frost resistance and drought resistance, we used phylogenetic mixed models. We used a large published seed plant phylogeny (Smith & Brown, 2018), which we matched to the species of our trait database with R packages ape (Paradis *et al*., 2004) and phytools (Revell, 2012). Our trait dataset had 236 species not present in the original tree: We placed them within their genera at a random place using the ‘add.species.to.genus’ function from the phytools package. This resulted in a final phylogeny with 1304 tips. We then pruned tips from the tree to match it with species for which both resistance traits were present in the database, resulting in a matching tree and dataset with 213 species. We also made trees matching each of the embolism resistance dataset (851 species) and the frost resistance dataset (457 species). For both traits, we computed phylogenetic signal statistics, using the phylosig function of the phytools package. We calculated both Blomberg's *K* (Blomberg *et al*., 2003) and Pagel's λ (Pagel, 1999). These statistics aim to quantify the degree of similarity in traits between closely related species, relative to a Brownian motion model of trait evolution. Values of K or λ close to 1 indicate strong phylogenetic signal – closely related species share similar trait values. On the other hand, values of K or λ close to 0 indicate low phylogenetic signal, with related species less similar than expected under Brownian motion. We used the built‐in tests to check for significant deviation from 0 for both statistics. For λ, we tested whether λ was different from 1 using a likelihood ratio test against a Brownian motion model of evolution. We did this using the phylosig function of the phytools package (Revell, 2012). Finally, for the tree with 213 species with data with both traits, we mapped the evolution of each trait using maximum likelihood estimation of ancestral states with contMap in phytools in R (Fig. S7).

We fit both phylogenetic and nonphylogenetic Bayesian mixed effects quantile regressions implemented in the Stan probabilistic programming language (Carpenter *et al*., 2017), thanks to the brms package in R (Bürkner, 2017, 2018). We modeled the relationship between embolism resistance and frost resistance with the functional group (deciduous angiosperms, evergreen angiosperms and gymnosperms) as a grouping variable with a separate slope and intercept per group. In a second set of models, we included the phylogenetic covariance matrix to model covariation across species based on shared evolutionary history. Finally, to test whether the strength and direction of the inferred relationships change in different parts of the data distribution, we fit quantile regressions in this framework, specifically at the 0.1, 0.5 (i.e. the median) and 0.9 quantiles. This is implemented in Stan using the asymmetric Laplace distribution. The Hamiltonian Monte‐Carlo Sampler achieved good convergence over 10 000 iterations, with 2000 discarded as burn‐in, based on ESS (Effective Sample Size), Rhat and visual inspection of the chains.

To test for a significant relationship, we compared the posterior distribution of the β‐coefficients (95% high‐density intervals (HDI)) – that is the regression slopes – to the ‘region of practical equivalence’ (ROPE) (Kruschke & Liddell, 2018), based on ±0.1SD of the y variable (Kruschke, 2018).

Data preparation and statistical analyses were made in R (R Core Team, 2023), using packages ape (Paradis *et al*., 2004), phytools (Revell, 2012) and geiger (Pennell *et al*., 2014) for phylogeny‐related tasks, and tidyverse (Wickham *et al*., 2019). We also used R code provided in Vargas *et al*. (2022) for comparing models.

## Results

### Drought vs frost resistance trade‐off

**Fig. 1:**
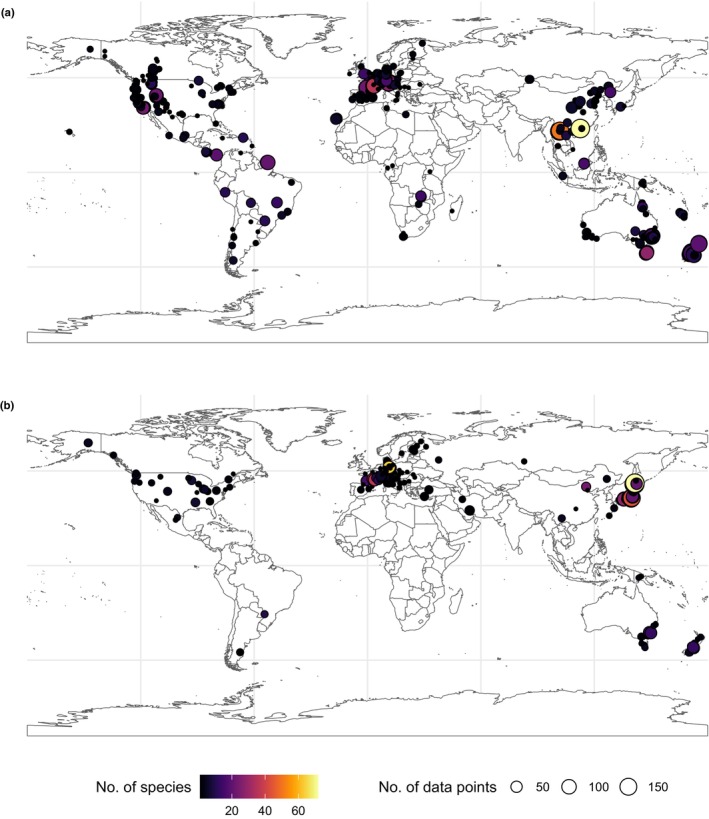
Data distribution of (a) the drought resistance and (b) frost resistance databases. Coordinates represent the study site or population of origin of the plants, when that information was available. The color of the points represents the number of different species, and circle size highlights the number of individual data points summarized across all species, for each cell of 1° latitude by 1° longitude.

**Fig. 2:**
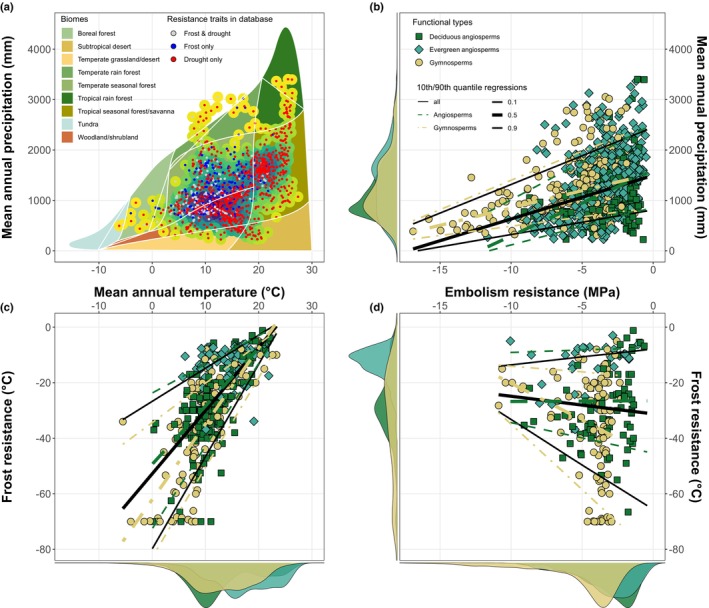
Functional trait trade‐off of frost and drought stress resistance and their relationship with the climate ranges of tree species. (a) Mean annual precipitation (MAP) and temperature of the ranges of species in the trait database (*n* = 1023). Shaded areas represent the Whittaker biomes (adapted from Ricklefs, 2008). Large dots represent the density of overlapping species on the graph (yellow = low density, darkblue = high density). Small dots show which stress resistance traits are present in the database used in this study. Relationships between (b) drought‐induced embolism resistance (P_50_ in MPa) and MAP (mm), and (c) frost resistance (LT_50_) and mean annual temperature (°C). (d) Trade‐off between drought‐induced embolism resistance and frost resistance. Colors and symbols indicate (dark‐green squares) deciduous and (blue‐green diamonds) evergreen angiosperms and (yellow circles) gymnosperms. Lines in (b–d) show quantile regression models for angiosperms (dashed line, green), gymnosperms (dot‐dashed line, yellow) and all species together (solid black line).

Our trait dataset covered all terrestrial biomes in which trees live (Figs 1, 2a), with MAP varying from *c*. 200 to over 3000 mm yr^−1^, and mean annual temperature from −5°C to over 20°C (Fig. 2a). Reflecting this climatic gradient, our newly developed database highlights the broad range of frost resistance (LT_50_) across woody species, from slightly below 0°C down to −80°C in the most resistant species. Species in cold environments are more resistant to frost (Fig. 2). There is large variability of LT_50_ at a given mean annual temperature, but frost resistance tracks more closely the lowest minimum daily temperature (Fig. S6), with gymnosperms slightly overadapted (mostly below the 1 : 1 line) and angiosperms more vulnerable (above the 1 : 1 line). Furthermore, evergreen angiosperms tend to be more frost‐tender, with higher average LT_50_, and a lower slope of the relationship with minimum temperature (Fig. S6). Frost resistance is also correlated with the United States Department of Agriculture maximum hardiness zone classifications, highlighting the strong adaptive nature of this trait and its usefulness for describing species range limits at the cold edge of the distribution (Fig. S8).

**Fig. 3:**
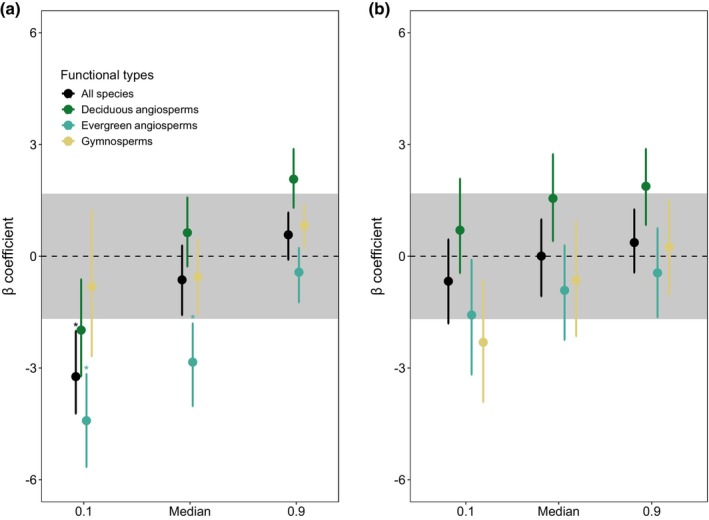
Posterior distribution (median ± 95% HDI) of regression slopes (β‐coefficients) from (a) nonphylogenetic and (b) phylogenetic Bayesian mixed effects quantile regressions between embolism resistance and frost resistance at quantiles 0.1, 0.5 and 0.9. The shaded area represents the Region of Practical Equivalence, indicating where the slopes are not significantly different from 0. Black symbols represent the regression analyses across all species, while the colors show group‐specific slopes for deciduous (yellow) and evergreen (light blue) angiosperms, and gymnosperms (dark green).

Embolism resistance (P_50_) varied from extremes of close to −19 MPa in some Australian conifers (Larter *et al*., 2015) to above −1 MPa in species from wet tropical environments (Fig. 2). Gymnosperms and evergreen angiosperms tend to be more embolism‐resistant and have a wider range of P_50_ than deciduous angiosperms. We found a strong link between high embolism resistance and low rainfall environments (Figs 2b, S6), whereas the more vulnerable species are restricted to wet environments. These findings are consistent with previous interspecific analyses across wide taxonomic ranges (Maherali *et al*., 2004; Choat *et al*., 2012; Hammond *et al*., 2021). There was no strong correlation between P_50_ and LT_50_ (Fig. 3). However, drought‐resistant species tend to be less frost hardy, and frost‐resistant species tend to be vulnerable to drought (Fig. 2c). There are notably few species extremely resistant to both stresses, and the ones reaching the lower limit of this trait space tend to be shrubs with slow growth, such as junipers.

The Bayesian quantile regression models show that although there was no significant trend at the median (quantile 0.5) of the trait distribution except in evergreen angiosperms, at lower quantiles (0.1) of the trait distributions, that is when either resistance trait is at extreme values, the regression slopes (β coefficients; Fig. 3a) leave the ROPE, indicating they are no longer equivalent to 0. This shows that extreme resistance traits are acting as constraints on the evolution of one another, especially in conifers and evergreen angiosperm species (Fig. 3). Incorporating the phylogenetic correlation structure into the regression model strongly reduced the slope of the regressions, with only the slope in gymnosperms mostly leaving the ROPE (Fig. 3b). This seems to indicate that a large part of the covariation between the traits is linked to phylogenetic structure in the data. Accordingly, we found P_50_ and LT_50_ to both display strong phylogenetic signal, with K and λ significantly different from 0 (Table 1). This indicates that closely related species are more similar in terms of stress resistance than expected by chance alone. However, λ is significantly different from 1, and K values are low, suggesting lower similarity between closely related species than based on a Brownian motion model of trait evolution (random walk along phylogenetic branches). This is consistent with adaptive traits that are strongly related to species' environmental preferences.

**Table 1:** Phylogenetic signal for embolism resistance and frost resistance.

Trait	Method	Phylogenetic signal	*P* vs 0	*P* vs 1	*n*
P50	*K*	0.032	0.001		1068
LT50	*K*	0.024	0.019		458
P50	λ	0.887	0	0	1068
LT50	λ	0.505	0	0	458

Blomberg's *K* (Blomberg *et al*., 2003) and Pagel's λ (Pagel, 1999) statistics were calculated using the phylosig function of the phytools package (Revell, 2012). *n* = number of species in each analysis. *P* vs 0 = *P*‐value based on 1000 tip permutations for *K* and a log‐likelihood ratio test against λ = 0. *P* vs 1 is a likelihood ratio test against a Brownian motion model of trait evolution fit using the fitContinuous function of phytools (i.e. significantly different from λ = 1).

This trend is confirmed by looking at the mapping of trait evolution across the phylogeny, with in general similar trait values across closely related species (Fig. S7), for example pines for P_50_ and maples for LT_50_, and *Quercus* or *Nothofagus* for both traits. On the other hand, in some clades, there is substantial variation in traits over relatively short evolutionary timescales – that is *Juniperus* for P_50_ and *Betula* for LT_50_. Furthermore, independent instances of evolution of extreme resistance to embolism, for example *Rosmarinus*, *Juniperus*, and frost hardiness, for example *Betula*, *Populus*, highlight the remarkable convergent evolution of these traits across large evolutionary timescales.

It is worth noting that although our data cover a wide range of biomes, we notably have lower coverage in warmer climates for frost resistance, for example from subtropical deserts to tropical forests (blue and gray dots in Fig. 1a) where live many drought‐adapted clades at the extremes of the P_50_ spectrum. We know however that many embolism‐resistant conifers are frost‐sensitive; for example, *Callitris* and *Cupressus* are mostly only hardy to USDA Zones 8–10 (Bannister & Neuner, 2001). Most of the 213 species with data for both traits occur in temperate biomes in the precipitation range between 500 and 2000 mm yr^−1^ and with mean annual temperatures between +5 and +15°C (woodland/shrubland and temperate seasonal forests). However, the dataset contains a number of species whose ranges extend into regions with very cold winters, that is minimum temperatures below −25°C and regions in the arid to semiarid zones, that is with an AI below 0.5 (Fig. S9).

### Lack of trade‐off between resistance and growth/resource acquisition traits

**Fig. 4:**
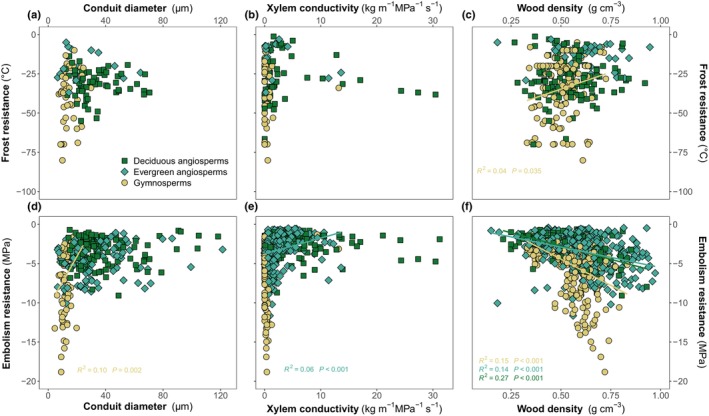
Relationships between xylem functional traits and climate resistance traits. Upper panels show frost resistance, lower panels show embolism resistance, and from left to right (a, d) xylem average conduit diameter (μm), (b, e) xylem‐specific hydraulic conductivity (kg MPa ^−1^ m^−1^ s^−1^) and (c, f) wood density (g cm^−3^). Colors and symbols show functional groups: deciduous angiosperms (green squares), evergreen angiosperms (blue‐green diamonds) and gymnosperms (yellow circles). Significant relationships at *P* < 0.05 are shown in each panel. To improve readability of (a, c), we do not show *Rhipidocladum racemiflorum*, a tropical bamboo with conduit diameter close to 200 μm.

We did not find a correlation between frost hardiness and either conduit diameter or maximum xylem‐specific conductivity (Kmax) in angiosperms (Fig. 4a,b), although a weak yet significant one exists in gymnosperms – with increasing wood density and increasing vulnerability to frost (Fig. 4c).

Wood density showed significant positive trends with embolism resistance (Fig. 4f). However, we found no relationship between conduit diameter or *K*_max_ and P_50_ – that is no safety–efficiency trade‐off, except for slight trends in conifers and evergreen angiosperms for each, respectively (Fig. 4d,e).

**Fig. 5:**
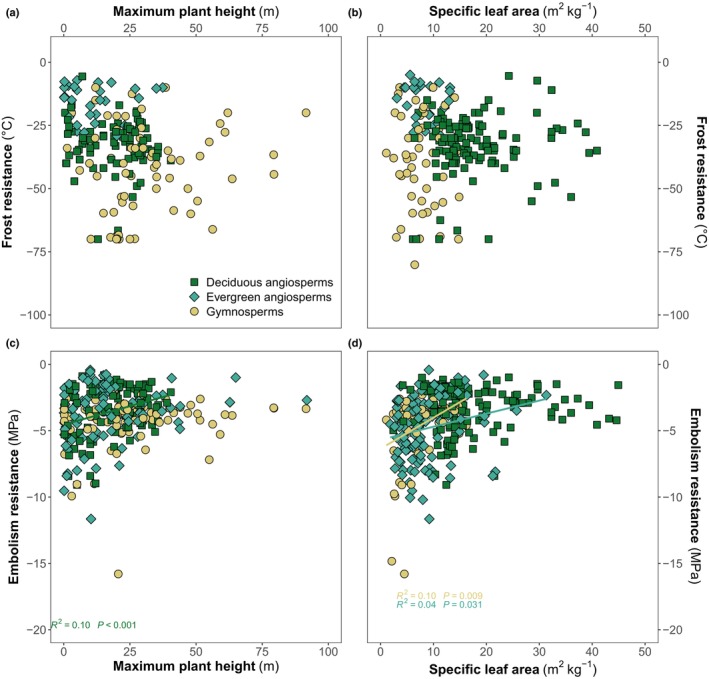
Plant maximum height (m) and specific leaf area (m^2^ kg^−1^) decrease with increasing embolism resistance (MPa) but there is no association with frost resistance (°C). Colors and symbols show functional groups: deciduous angiosperms (green squares), evergreen angiosperms (blue‐green diamonds) and gymnosperms (yellow circles). Significant relationships at *P* < 0.05 are shown in each panel.

We found no significant associations between growth traits, that is maximum height and specific leaf area (SLA) and frost resistance (Fig. 5a,b). Especially in gymnosperms, there are species that can grow very tall yet display high frost resistance, and the smallest species cover the whole range of frost resistance. For SLA, as expected, evergreen angiosperms and conifers have the toughest leaves, while deciduous angiosperms have higher SLA and a broader range. However, no trend with frost resistance is evident in any of these groups.

On the other hand, embolism‐resistant species tend to be shorter, although the relationship is significant only in deciduous angiosperms (Fig. 5c), and have smaller, tougher leaves at least in deciduous angiosperms and gymnosperms (Fig. 5d). Furthermore, for drought resistance, it seems there is an area of trait space unexplored by species in our dataset: There are no embolism‐resistant trees that can grow extremely tall or are able to deploy a large leaf area for a given biomass investment. This supports the hypothesis that tree growth rates can be limited by increased embolism resistance.

Leaf area (mm^2^) followed a similar pattern to SLA, that is no significant relationship with frost resistance and a weak trend toward smaller leaves in embolism‐resistant species (Fig. S10).

## Discussion

### Main conclusions

Through this unique trait database with broad sampling across all forested biomes, we found evidence of a weak trade‐off between frost and drought resistance. In support of our main hypothesis, we show that woody species that have evolved strong resistance to one type of stress tend to be vulnerable to the other type of stress, and few species have been able to evolve high resistance to both frost and drought. On the other hand, many species are relatively vulnerable to both stresses, indicating that when selective pressure for stress resistance is relaxed in biomes with only mild summer drought and relatively mild winters, plants optimize along other axes of fitness. The quantile regression analysis showed that at the lower quantiles, there is a significant and stronger regression slope, which indicates constraints on drought resistance in cold environments and vice versa. However, including the phylogeny reduced the significance of this effect, reflecting the influence of deep phylogenetic divergences in the evolution of these traits. This is confirmed by the significant phylogenetic signal for both traits. We investigated potential costs or trade‐offs of resistance traits with regard to growth and competition (Hypothesis 2), but we did not find any strong effects of frost resistance on xylem efficiency, wood density, maximum height or specific leaf area. By contrast, increased xylem embolism resistance generally comes at the cost of denser wood with smaller conduits, as well as shorter stature with smaller leaves.

### Resistance traits trade‐off

Quantile regressions are an interesting tool for investigating evolutionary constraints when we expect the strength of the relationships to increase in extreme conditions. In these cases, looking at extreme quantiles of the trait distributions can reveal constraints that are obscured around the mean or median of the trait distribution, using traditional means‐based statistical approaches such as ordinary least squares regressions (Cade & Noon, 2003). Our results show that toward the limits of the distribution of frost and drought resistance in our dataset, constraints appear that limit variation of one trait relative to another, revealing a trade‐off. In other parts of the trait distributions, these constraints are not apparent, and other processes likely determine possible resistance trait combinations, such as light and nutrient availability. However, including the phylogeny in this quantile regression analysis reduces the strength of this result, indicating that part of the correlation is linked to phylogenetic relationships and not a functional link between frost and drought resistance traits. Because of the significant (but relatively low) phylogenetic signal for both resistance traits, we can expect that deep phylogenetic divergences (such as the split between angiosperms and gymnosperms) are responsible for this pattern (Ackerly & Reich, 1999; Ávila‐Lovera *et al*., 2023).

There are great differences in the structure and function of xylem between angiosperms and gymnosperms (Sperry *et al*., 2006). It is known that on average, angiosperms tend to be less resistant to drought‐induced xylem embolism than gymnosperms (Augusto *et al*., 2014), likely because of differences in the interconduit pits (Hacke *et al*., 2005; Sperry *et al*., 2006). The torus of gymnosperms' heterogeneous pit membranes acts as a valve that limits the spread of air bubbles (Delzon *et al*., 2010). In angiosperms, the thickness of the membrane limits the spread of air bubbles, with species with thicker membranes being more resistant to embolism (Li *et al*., 2016; Thonglim *et al*., 2021). For frost resistance, the distribution of trait values largely overlaps (Fig. 2), with evergreen angiosperms being less resistant than deciduous angiosperms. Gymnosperms show a much flatter distribution, with more extremely resistant species than angiosperms. Although vulnerability to freeze–thaw embolism of angiosperms is likely related to their wider xylem conduits (Zanne *et al*., 2014), the diversity of cell types in angiosperm wood, relative to gymnosperms (which are composed mostly of tracheids), could favor adaptation to freezing temperatures. Because of the distribution of modern conifers world‐wide and their apparent domination in high latitude and elevations and in poor sandy soils, it is thought that the better competitive ability of angiosperms in favorable conditions restricted gymnosperms to stressful habitats (Bond, 1989; Augusto *et al*., 2014). Gymnosperms certainly tend to dominate both extremes of the resistance traits presented in this paper, with the most frost‐resistant and drought‐resistant species almost exclusively made up of gymnosperms. Our results highlight the need to further study the drought‐ and frost‐tolerant strategies of species growing in dry and cold environments such as junipers (Larter *et al*., 2024).

To our knowledge, this is the first effort to combine woody plant drought‐induced embolism resistance and frost resistance in a global database. For embolism resistance, previous work has aimed to compile data from the literature (Choat *et al*., 2012; Hammond *et al*., 2021), focusing on the evolution of embolism resistance, the vulnerability of tree species to climate‐change‐induced drought (Sanchez‐Martinez *et al*., 2023) and relationships with functional traits linked to the xylem such as wood density and water transport efficiency (Gleason *et al*., 2016). An interesting study highlighted the widespread occurrence of areas vulnerable to both summer drought and winter freeze–thaw events, but without including frost resistance traits (McCulloh *et al*., 2023). For this trait, previous efforts have been focused on narrower taxonomic or geographic scales (Sakai & Weiser, 1973; Bannister & Neuner, 2001). A recent study examined the global distribution of thermal tolerance, combining datasets for extreme hot and cold temperature cellular damage (Lancaster & Humphreys, 2020). How hot droughts kill trees is increasingly studied (e.g. Mantova *et al*., 2023), and efforts to combine multiple facets of drought mortality in an evolutionary framework will likely be very interesting. This study built on these previous efforts, including evaluating the vulnerability of some data to potential artifacts based on measurement methods. More efforts generally in the field are required to highlight pitfalls and avoid publishing inaccurate data. In this paper, we took appropriate steps to remove as many of these erroneous data points as possible, and urge scientists to apply strict quality control for example when embolism resistance values (typically P_50_ or P_88_) are above the turgor loss point, for a given species. Some methods have known issues in long‐vesseled species and should be cross‐validated with a different method that does not require cutting the sample (Torres‐Ruiz *et al*., 2015), for example using μCT or the optical technique. For functional traits that are difficult or time‐consuming to measure, using modeling approaches to infer trait values from correlation structures and phylogenetic relationships is a new approach that we did not explore here (Sanchez‐Martinez *et al*., 2023). The lack of very strong predictive relationships between each resistance trait and climate or other traits induces the risk of large errors in the predicted trait data, especially in lineages with very long branches or rapid evolution rates. Instead, we chose to add new data through additional measurements and produced new frost resistance data for 80 species, and embolism data for 15 species (Supporting Information). While this paper combines two of the most studied traits related to drought and frost resistance, these traits are not perfect to describe a species' stress resistance – but see baranger_living_2024. Embolism resistance is only one facet of drought response; our approach could be improved by including more traits such as the water potential at turgor loss (Ψ_TLP_), or residual leaf and bark water loss at a particular water potential (g_min_ and g_bark_) in a multivariate framework (Cochard *et al*., 2021; Petek‐Petrik *et al*., 2023). We could also work to exclude species that escape the need for embolism resistance, for example by increasing rooting depth (Laughlin *et al*., 2023). Additionally, while variation in P_50_ is often small within species (Lamy *et al*., 2011; Bouche *et al*., 2016; Lobo *et al*., 2018; Alon *et al*., 2023), there exists variation in some species, within the plant based on organ age or growth rate differences (Sorek *et al*., 2022; Johnson & Brodribb, 2023; Grossman *et al*., 2024), or across populations (López *et al*., 2013; Schuldt *et al*., 2015; Stojnić *et al*., 2018). Depending on the phylogenetic scale under consideration, including more measurements per species and accounting for within‐species phenotypic variation explicitly in models could be important, as the evolution of these traits over a short timescale seems possible in some clades (Larter *et al*., 2017) but not in others (Lamy *et al*., 2011).

Similarly, frost resistance is a seasonally dynamic trait, and plants tend to be more sensitive to early frost, that is before hardening, or after bud break, when selective pressure for frost resistance is highest (Lenz *et al*., 2013; Charrier *et al*., 2013b). Yet, because the hardening process varies across species, to make meaningful comparisons across species, it is best to compare frost resistance at maximum (i.e. full winter) hardiness. This trait has been shown to be predictive of species distributions (Baranger *et al*., 2024). Warm spells just before fieldwork campaigns can trigger dehardening, and this information is hard to quantify, especially from the literature (Kalberer *et al*., 2006). As for embolism resistance, there is likely intraspecific variation for this trait, but this varies between species (Harbol *et al*., 2023). In our dataset, we found little evidence for this, or for plasticity or acclimation of tissues after (repeated) exposure to extreme negative temperatures (Larcher & Mair, 1968). Quantifying this response in detail requires repeated measurements across a range of dates and environmental conditions, making it notoriously difficult. Our approach using mid‐winter maximum hardiness, which should be well‐correlated with spring (and fall) frost resistance is likely the best compromise between attaining accurate estimates of a species' resistance, maintaining comparability across species and preserving the mental health of the scientists involved. We chose the EL technique as a reference method, relative to other methods such as VS or differential thermal analysis, because of how quick and repeatable the measurements are following established standard protocols (Aronsson & Eliasson, 1970; Stergios & Howell, 1973). Furthermore, some conifer species are difficult to measure accurately using the EL technique, especially in species with extreme frost resistance. Obtaining high levels of cell mortality in these species requires the use of liquid N_2_, rendering the control of the rate of freezing and thawing of the samples elusive. In others, for example in genera *Larix* and *Picea*, getting accurate electrolyte measurements from small terminal twigs proved difficult. Perhaps, there are too few living cells in the samples in gymnosperms (where living cells are only in the rays) compared with angiosperms, or the presence of large amounts of resin adds noise to the electrolyte measurement data.

Finally, the traits under consideration describe the resistance of different plant compartments, which could explain the weakness of the trade‐off in this study. Frost resistance is typically measured on stem segments made up of cortical tissues and phloem, and living cells of the xylem, in contrast to xylem embolism resistance measurements, that focus specifically on the dead cell components of the vascular system. Put another way, frost resistance as described in this work relates to the symplastic failure of the living plant tissues, whereas drought resistance (xylem embolism resistance) deals with apoplastic hydraulic failure. In the hot‐drought death sequence, apoplastic hydraulic failure occurs before symplastic failure of living cells (Mantova *et al*., 2023). Frost damages occur upon freezing for symplastic failure, but upon thawing for apoplastic failure (freeze–thaw embolism) (Sakai & Larcher, 1987b). Therefore, the sequence and consequences of the stresses are different when considering the cellular or extracellular water compartments.

### Life‐history strategies and trade‐offs

Trait combinations of plants are driven by life‐history strategies to optimize resource capture and storage, growth, reproduction and survival in a given environment (Grime, 2006). The classic CSR (Competitive, Stress‐tolerant, Ruderal) framework places species on varying stress and disturbance axes, classifying species based on their tolerance to low/high stress and low/high levels of disturbance (Grime, 1977, 2006). In this framework, stress‐tolerating species are typically viewed as slow‐growing and have typically conservative traits such as small evergreen leaves and low reproductive output. On the opposite end of the stress resistance spectrum, competitive species tend to favor high growth rates and resource acquisition traits (such as large tender leaves with a short lifespan), which is consistent with other approaches to plant trait optimization frameworks, such as the growth–mortality trade‐off (Russo *et al*., 2021) or the fast vs slow plant economics theory (Reich, 2014). One limit of these frameworks is that all stresses are placed on the same axis, which we try to untangle in this study. We hypothesized that our results would reflect species strategies and their ranking along the stress tolerance axes and that species vulnerable to climatic stresses should be optimizing growth or resource acquisition traits. However, we found few significant relationships between resistance traits and growth or competition traits, as represented by the absence of significant trends between frost resistance and maximum plant height, specific leaf area, xylem water transport efficiency and wood density. However, high resistance to xylem embolism is associated with having smaller conduits (especially in gymnosperms), and denser wood. This compromise does not necessarily result in low hydraulic efficiency, as species with low embolism resistance can have both low‐ and high‐density wood, and low and high efficiency xylem, which is consistent with previous results (Gleason *et al*., 2016; Larter *et al*., 2017). Rather, we found no dependence of frost resistance on conduit size, which goes against the idea that species from cold environments should evolve smaller conduits to avoid freeze–thaw embolisms. This is consistent with the observation that wider vessels do not necessarily embolize first (Charra‐Vaskou *et al*., 2016, 2023). We found a slight trend to lower density wood in frost‐resistant conifers, but the relationship is very weak, and no trend exists in either evergreen or deciduous angiosperms. This agrees with other work finding a disconnection between purely hydraulic survival‐related traits and growth in both herbs and woody plants and at different geographical and taxonomic scales (Maréchaux *et al*., 2015; Blackman *et al*., 2016; Li *et al*., 2018; Yin *et al*., 2018; Ramírez‐Valiente *et al*., 2020; Rosas *et al*., 2021; Huang *et al*., 2025). This further suggests the likely co‐occurrence of different independent axes of natural selection able to at least in some species and climates drive the variation of these traits in a number of directions, sometimes parallel but not always. As stated before, the functional traits included here do not cover all fitness components or strategies, and other axes of investigation for example regarding shade tolerance or reproduction could discover other critical trade‐offs.

### Conclusion

We found a weak trade‐off between drought and frost resistance traits across a broad species‐level database, covering all forested biomes. While there are a number of species vulnerable to both stresses, others have developed high resistance to either drought or extreme cold temperatures, but never both. Using quantile regressions, we show evolutionary constraints increase the slope of the trade‐off relationship when one trait value is at extreme parts of the distribution. These constraints are released for moderate and low trait values, indicating other factors could be acting as constraints on resistance traits. However, we have not identified a limiting trade‐off for frost resistance based on the traits studied here, offering avenues for future research incorporating additional traits and potentially coordinated trait responses. Our results highlight the risk to forests in cold biomes with drying summers, and dry environments with more unpredictable winter warm spells and late frosts, since few species have evolved resistance to both stresses.

## Competing interests

None declared.

## Author contributions

ML wrote the initial draft, ran the analyses and made the figures. GK, SD and GC initiated the project. Sample collection and trait measurements resulted from a collective effort by ML, GK, GC and AB. SD, CB, NM‐S and WH provided data. All authors contributed to the final manuscript.

## Disclaimer

The New Phytologist Foundation remains neutral with regard to jurisdictional claims in maps and in any institutional affiliations.

## Supporting information


**Fig. S1** Comparison of frost tolerance methods: visual scoring (LT_0_) vs electrolyte leakage (LT_50_).
**Fig. S2** Comparison of frost tolerance data across the different organs in the database, classified as bud, branch and leaf for both LT_0_ and LT_50_.
**Fig. S3** Comparison of two datasets of frost tolerance, cleaned vs noncleaned.
**Fig. S4** Comparison of the different methods from the embolism resistance database.
**Fig. S5** Tree maximum height (m) comparison between data from the allometric models and from TRY/Tallo databases.
**Fig. S6** Extreme climate – minimum temperatures and aridity index – and resistance trait variation.
**Fig. S7** Evolutionary history of P_50_ (left side) and LT_50_ (right side) on a phylogeny of 186 tree species in the trait database.
**Fig. S8** Regression of frost tolerance data vs species USDA maximum hardiness zone.
**Fig. S9** Distributions of species and resistance traits based on climatic extremes: 5^th^ percentile of the species range of aridity index and minimum temperature.
**Fig. S10** Frost tolerance is not related to leaf area (mm), however, more embolism‐resistant angiosperms tend to have smaller leaves (not significant in gymnosperms).
**Methods S1** Additional methods section detailing the methods used for the new measurements presented in this study for species frost and drought resistance.

**Table S1** New measurements of frost tolerance for the purpose of this study.

**Table S2** New measurements of embolism resistance for the purpose of this study.

**Table S3** Full trait and climate data (including sources).
Please note: Wiley is not responsible for the content or functionality of any Supporting Information supplied by the authors. Any queries (other than missing material) should be directed to the *New Phytologist* Central Office.

## Data Availability

The data that supports the findings of this study are available in the Supporting Information of this article. New measurements for frost and drought resistance are in Tables S1 and S2, respectively, and the full species‐level trait data and relevant references (for previously published data) are in Table S3.
