# Supplementary material for: Weak global trade‐off between frost and drought resistance in trees
Source: New Phytol. 2025 Nov 9;249(2):810–28. doi: 10.1111/nph.70718 (PMC12712442; doi:10.1111/nph.70718)
Supplement: Supplementary file 1 — Fig. S1 Comparison of frost tolerance methods: visual scoring (LT0) vs electrolyte leakage (LT50). Fig. S2 Comparison of frost tolerance data across the different organs in the database, classified as bud, branch and leaf for both LT0 and LT50. Fig. S3 Comparison of two datasets of frost tolerance, cleaned vs noncleaned. Fig. S4 Comparison of the different methods from the embolism resistance database. Fig. S5 Tree maximum height (m) comparison between data from the allometric models and from TRY/Tallo databases. Fig. S6 Extreme climate – minimum temperatures and aridity index – and resistance trait variation. Fig. S7 Evolutionary history of P50 (left side) and LT50 (right side) on a phylogeny of 186 tree species in the trait database. Fig. S8 Regression of frost tolerance data vs species USDA maximum hardiness zone. Fig. S9 Distributions of species and resistance traits based on climatic extremes: 5th percentile of the species range of aridity index and minimum temperature. Fig. S10 Frost tolerance is not related to leaf area (mm), however, more embolism‐resistant angiosperms tend to have smaller leaves (not significant in gymnosperms). Methods S1 Additional methods section detailing the methods used for the new measurements presented in this study for species frost and drought resistance. [file NPH-249-810-s001.pdf]

New Phytologist Supporting Information

Article title:

Weak global trade-off between frost and drought resistance in trees

Authors: Maximilian Larter[1,2], Guillaume Charrier[2], Sylvain Delzon[1], William Hammond[3], Anne Baranger[4], Constance Bertrand[2,5], Nicolas Martin-StPaul[6], and Georges Kunstler[4]

[1]Univ. Bordeaux, INRAE, BIOGECO, 33600 Pessac, France
[2]Univ. Clermont Auvergne, INRAE, PIAF, 63000 Clermont-Ferrand, France
[3]Univ. of Florida, Gainesville, FL 32608, USA
[4]Univ. Grenoble Alpes, INRAE, LESSEM, 38400 St-Martin-d'Hères, France
[5]Univ. Rennes, CNRS, ECOBIO, 35700 Rennes, France
[6]URFM, INRAE, 84914 Avignon, France

Corresponding author: Maximilian Larter (maximilian.larter@inrae.fr)

Article acceptance date: 10 October 2025

List of Supplementary figures:

Fig. S1. Comparison of frost tolerance methods: visual scoring (LT0) vs. Electrolyte leakage (LT50).
Fig. S2. Comparison of frost tolerance data across the different organs in the database, classified as bud, branch and leaf for both Lt0 (A,C,E) and LT50 (B,D,F).
Fig. S3. Comparison of two datasets of frost tolerance, cleaned vs. non-cleaned.
Fig. S4. Comparison of the different methods from the embolism resistance database.
Fig. S5. Tree maximum height (m) comparison between data from the allometric models and from TRY/Tallo databases.
Fig. S6. Extreme climate - minimum temperatures and aridity index - and resistance trait variation.
Fig. S7. Evolutionary history of P_{50} (left side) and LT_{50} (right side) on a phylogeny of 186 tree species in the trait database.
Fig. S8. Regression of frost tolerance data vs species USDA maximum hardiness zone.
Fig. S9. Distributions of species and resistance traits based on climatic extremes: 5th percentile of the species range of aridity index and minimum temperature.
Fig. S10. Frost tolerance is not related to leaf area (mm), however, more embolism resistant angiosperms tend to have smaller leaves (not significant in gymnosperms).

List of Supplementary tables:

Suppl. Table 1: New measurements of frost tolerance for the purpose of this study.
Suppl. Table 2: New measurements of embolism resistance for the purpose of this study.
Suppl. Table 3: Full trait and climate data (including sources).

Supplementary Methods S1:

Additional methods section detailing the methods used for the new measurements presented in this study for species frost and drought resistance.

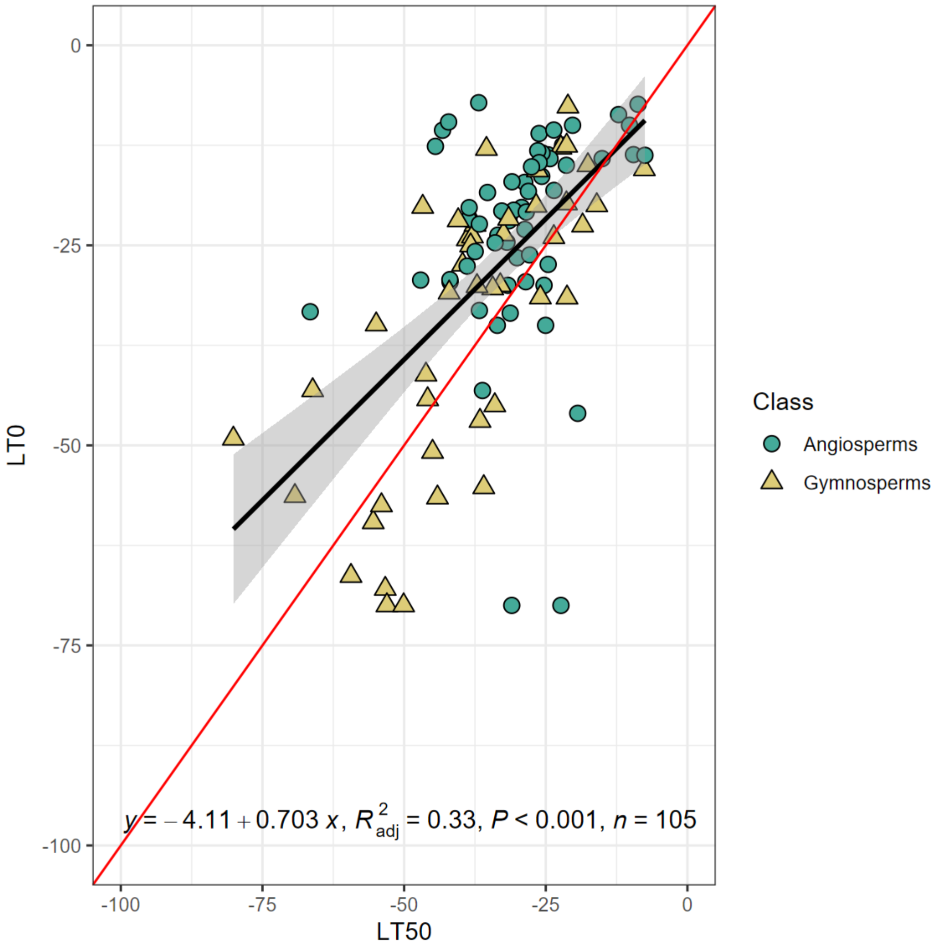

Figure 1. Comparison of frost tolerance methods: visual scoring (LT_0) vs. Electrolyte leakage (LT_{50}). Data shows points for the 110 species with data for both methods. Lines and shaded area show the linear model and 95% confidence interval, with the corresponding ajusted R-squared, p-value and number of points. Yellow triangles are for gymnosperm species and green dots for Angiosperms. Red line is the 1:1 line.

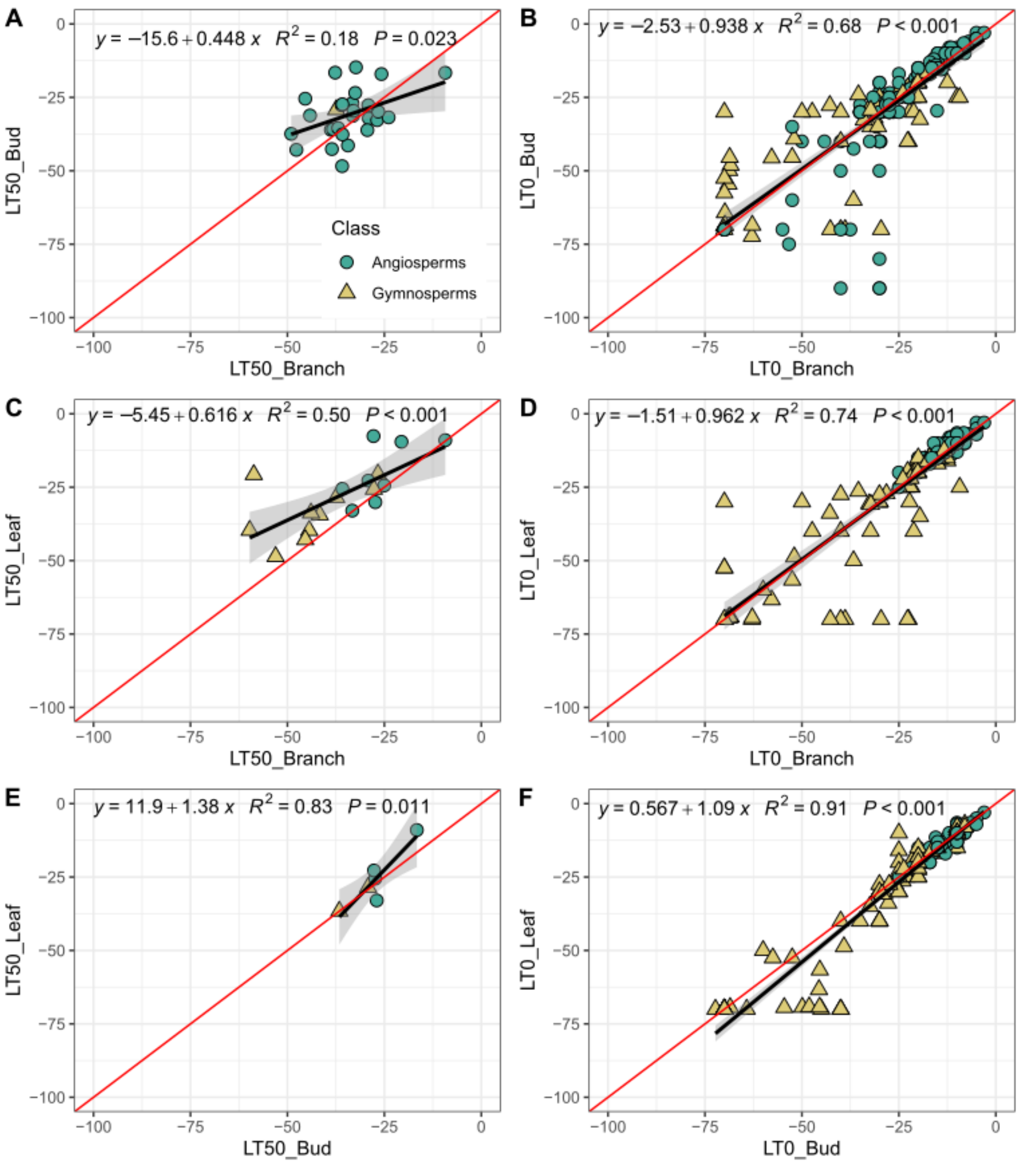

Figure 2. Comparison of frost tolerance data across the different organs in the database, classified as bud, branch and leaf for both LT_{50} (A,C,E) and LT_0 (B,D,F). In each panel, lines and shaded area show the linear model and 95% confidence interval, with the corresponding ajusted R-squared, p-value and number of points. Yellow triangles are for gymnosperm species and green dots for Angiosperms. Red line is the 1:1 line.

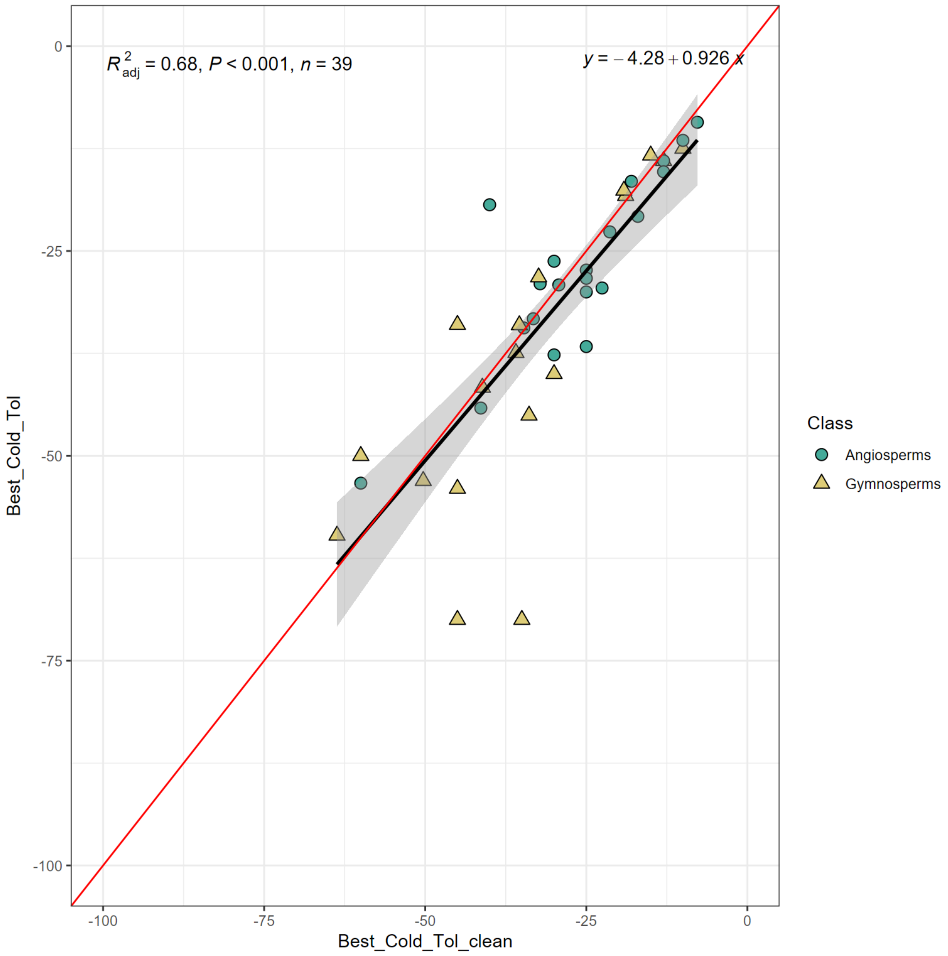

Figure 3. Species level comparison of the cleaned dataset of frost tolerance vs. the full database with juveniles and slow and/or rapid freezing and thawing rates. Lines and shaded area show the linear model and 95% confidence interval, with the corresponding adjusted R-squared, p-value and number of points. Yellow triangles are for gymnosperm species and green dots for Angiosperms. Red line is the 1:1 line.

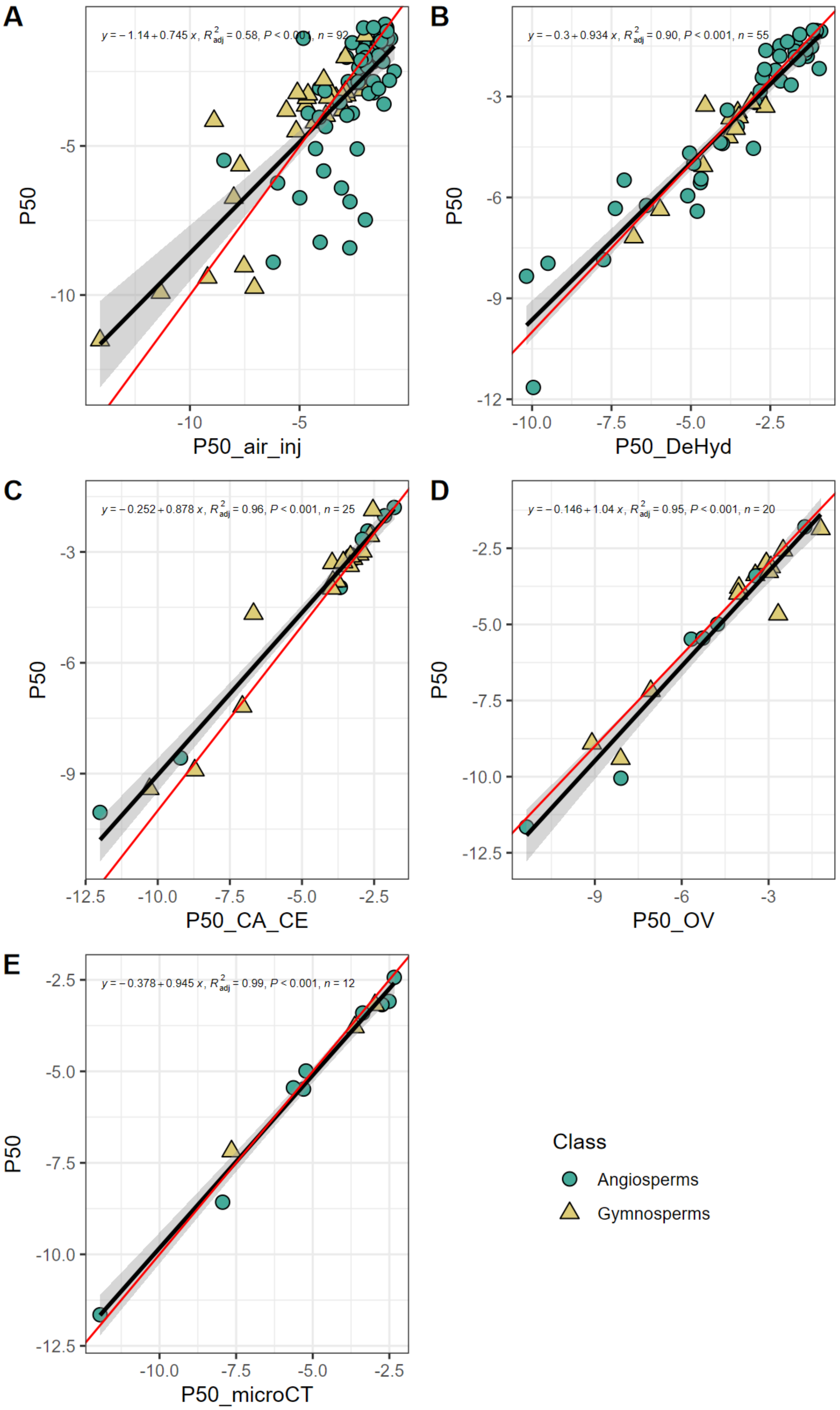

Figure 4. Comparison of the different methods from the embolism resistance database.

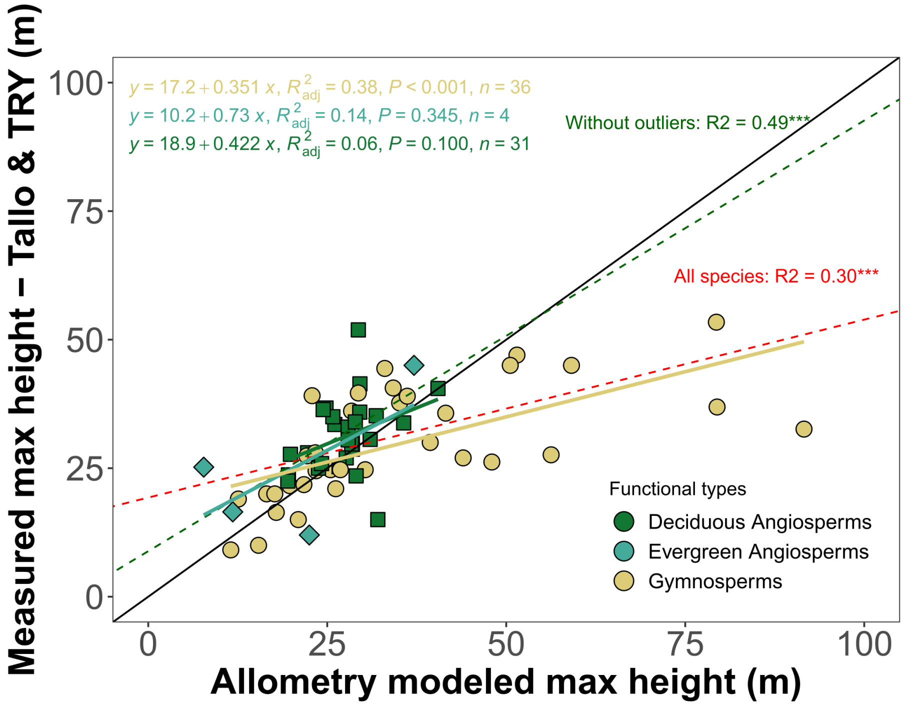

Figure 5. Tree maximum height (m) comparison between data from the allometric models and from TRY/Tallo databases.

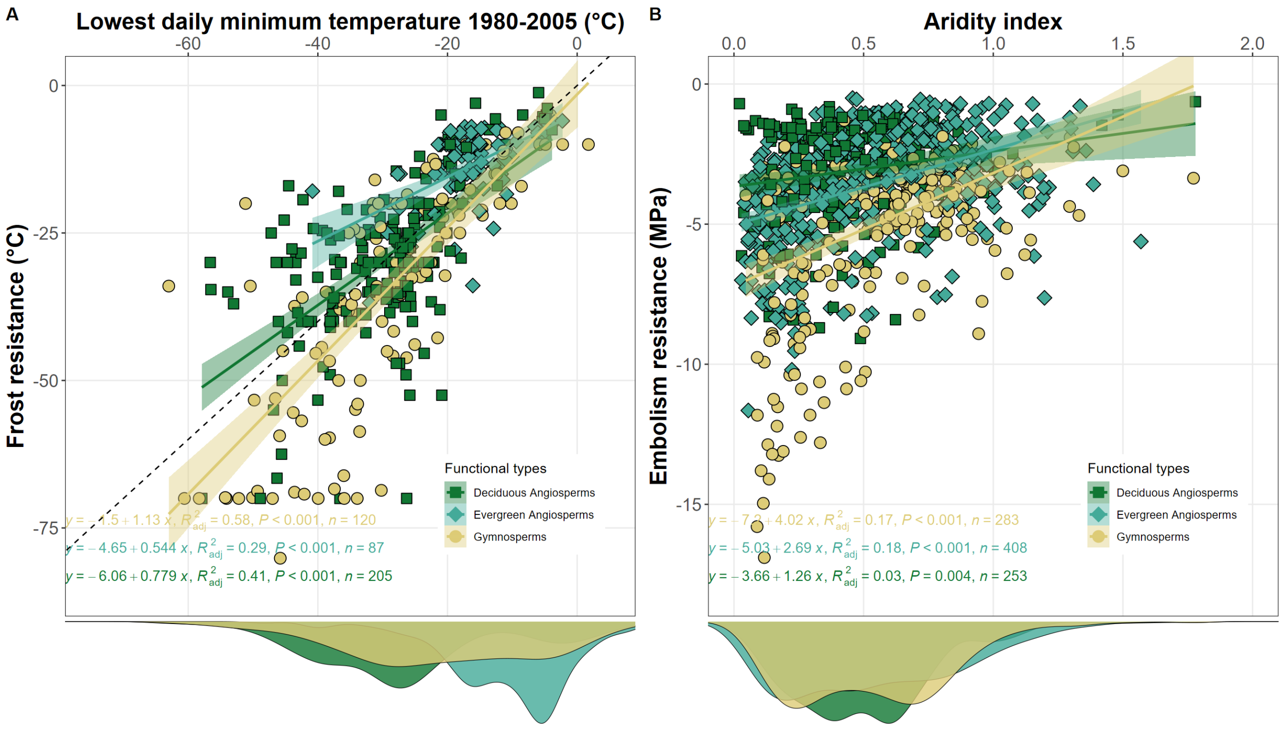

Figure 6. Extreme climate - minimum temperatures and aridity index - explain resistance trait variation. Each climatic variable represents for each species the 5th most extreme percentile of the range across GBIF occurrences.

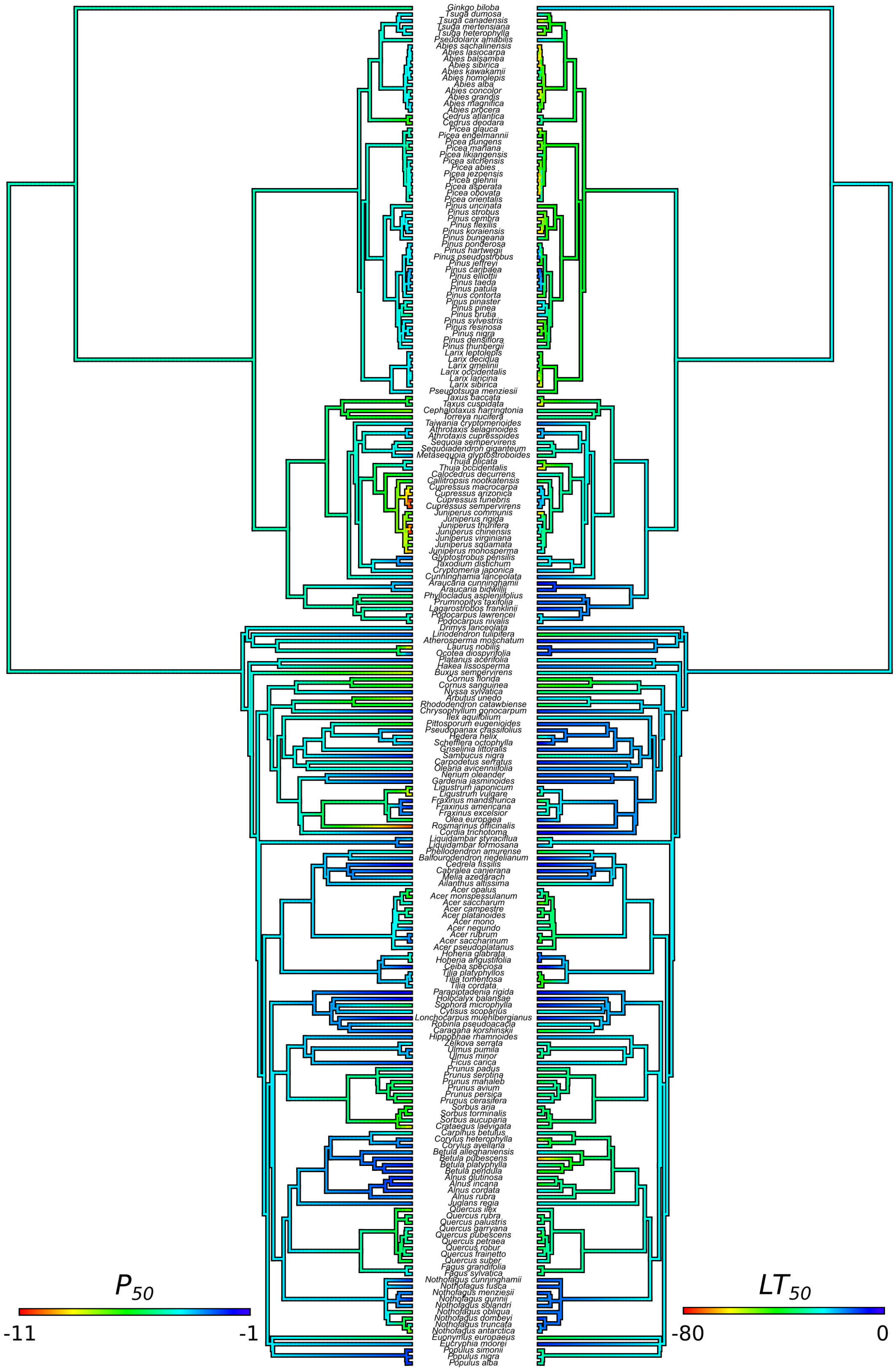

Figure 7. Evolutionary history of P_{50} (left side) and LT_{50} (right side) on a phylogeny of 213 tree species in the trait database. Trait evolution mapped using maximum likelihood estimation of ancestral states under a Brownian motion model of trait evolution, with red colors indicating higher resistance, and blue indicating vulnerability.

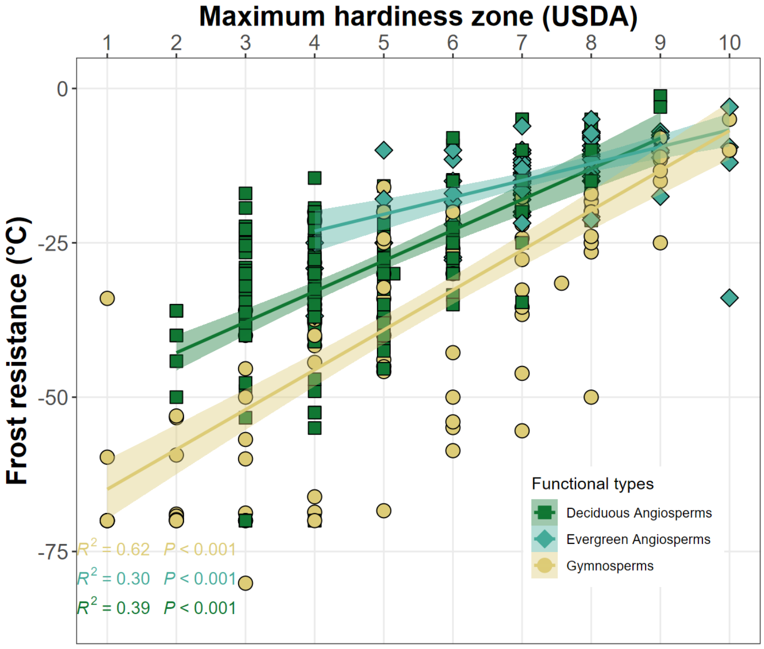

Figure 8. Regression of frost tolerance data vs species USDA maximum hardiness zone. The colored symbols and bold yellow, dark green and light green lines show linear regressions for gymnosperms and deciduous, evergreen angiosperms, respectively. Shading represents 95% confidence intervals and the corresponding linear model statistics are shown in the text on the figure.

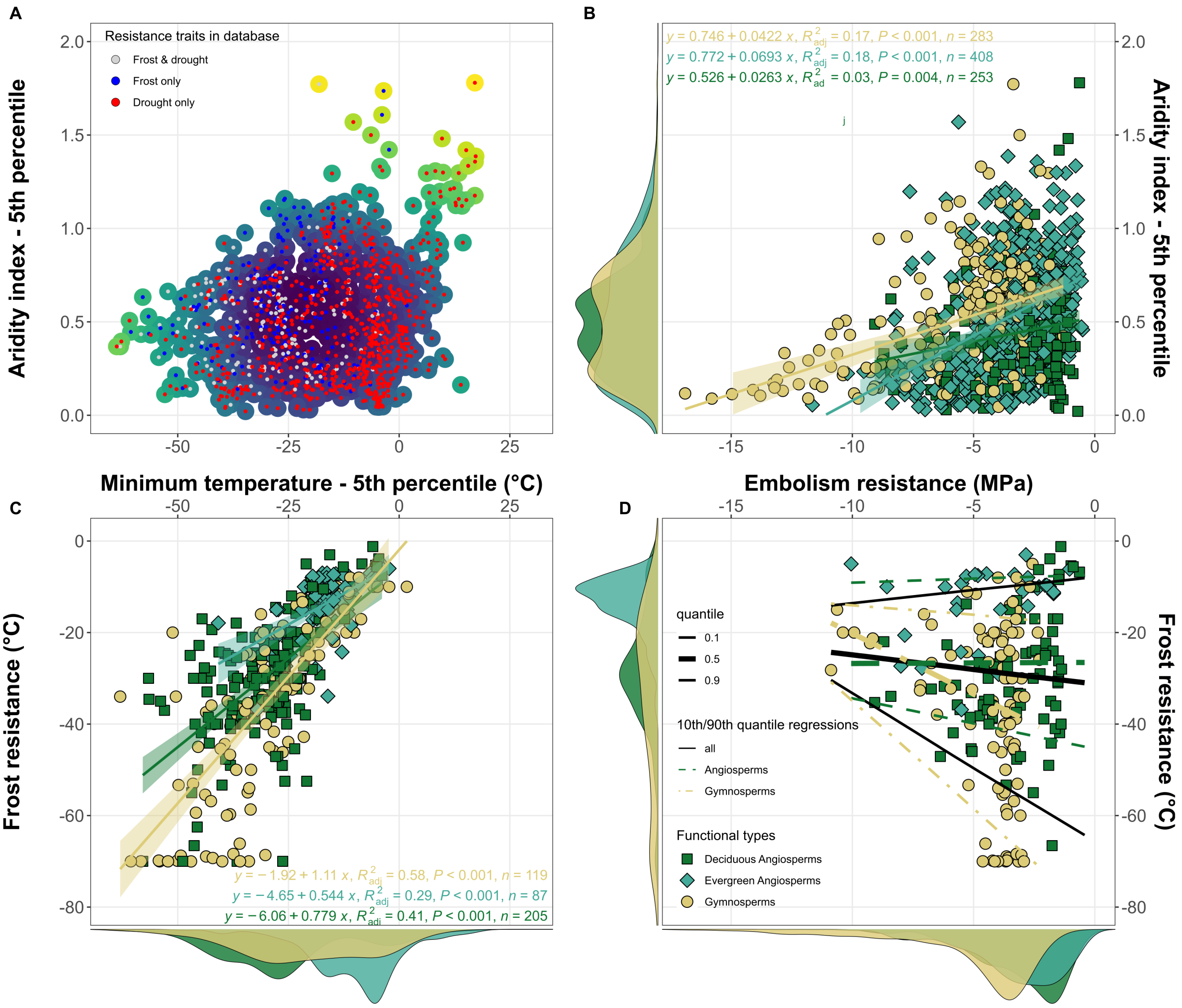

Figure 9. Resistance traits and the dry and cold limits of species distributions: 5th percentile of the species range of driest quarter precipitation and minimum temperature. (A) Driest quarter precipitation (mm) and minimum temperature at the 5th percentile of the ranges of species in the trait database (n = 1023). Large dots represent the density of overlapping species on the graph (yellow = low density, darkblue = high density). Small dots show which stress resistance traits are present in the database used in this study. Relationships between **(B)** drought-induced embolism resistance (P_{50} in MPa) and Driest quarter precipitation (mm), and **(C)** frost resistance and minimum temperature (℃). **(D)** Trade-off between drought-induced embolism resistance and frost resistance. Colors and symbols indicate (dark green squares) deciduous and (blue-green diamonds) evergreen angiosperms and (yellow circles) gymnosperms. Lines in panels B, C, and D show quantile regression models for angiosperms (dashed line, green), gymnosperms (dot-dashed line, yellow) and all species together (solid black line).

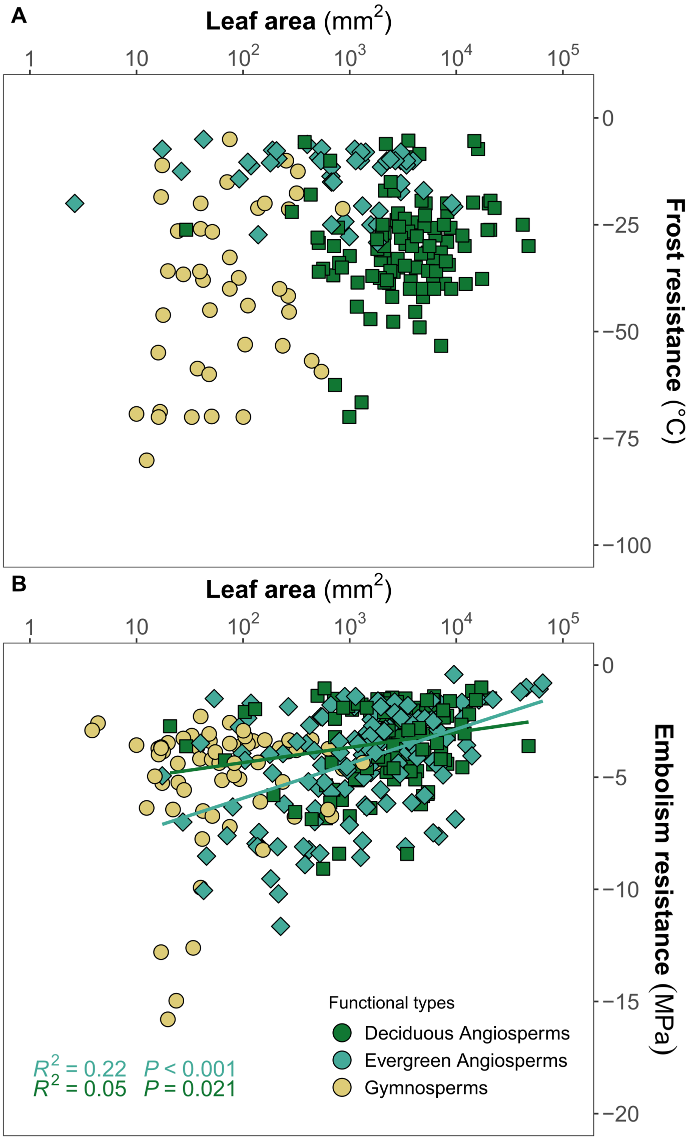

Figure 10. Frost tolerance is not related to leaf area (mm), however, more embolism resistant angiosperms tend to have smaller leaves (not significant in gymnosperms). The colored symbols and bold yellow, dark green and light green lines show linear regressions for gymnosperms and deciduous, evergreen angiosperms, respectively. Shading represents 95% confidence intervals and the corresponding linear model statistics are shown in the text on the figure.

Supplementary Methods S1

Frost resistance database

Naturally hardened tissue samples collected in mid-winter are exposed to a range of negative temperatures, and the amount of cellular damage is estimated by measuring the relative change in electric conductivity of the sample in pure water. The inflection point of the resulting sigmoid relationship between increasingly negative temperature and relative electrolyte leakage gives LT_{50}.

The electrolyte leakage technique (EL) employs a similar cold temperature gradient to the visual scoring (VS) technique, and quantifies the release of electrolytes from plant cells as they die following exposure to cold temperatures. From the sigmoid response curve of the relationship between the relative electrolyte leakage (REL; equation 1) obtained and the minimum temperature of exposure, we can derive the inflection point called LT_{50} (°C), i.e. the temperature at which 50% of REL is reached (equation 2).

We collected branches from 5 individual trees per species. Short stem segments from each individual tree were exposed to maximum negative temperatures of -15, -25, -35, -50, -80°C, as well as a +5°C control, and an extreme treatment using liquid nitrogen aiming to reach below -100°C for the most frost hardy species. The temperature dynamic was followed using thermocouples connected to a CR1000 datalogger (Campbell Scientific LTD, UK). For temperature settings between -15 and -50°C, we programmed four temperature-controlled chambers to drop by 5 K.h-1 from +5°C to the target temperature, hold for 1 hour at the target temperature, then return to +5°C at a rate of 5 K.h-1 . For the -80°C treatment, samples were placed in a large thermos container inside an -80°C freezer. Using a thermocouple, we monitored temperature inside the thermos which reached a minimum of around -70°C within a few hours. Thawing was achieved by placing the samples at room temperature ensuring gradual thawing over several hours. For the extreme treatment, liquid N_2 was placed at the bottom of an expanded polystyrene box. The samples were then placed on a platform and never in contact with the liquid N_2. A large temperature gradient existed between samples closest to the liquid, monitored with 5-10 thermocouple sensors placed throughout the box. The average minimum temperature across all thermocouples was used to estimate minimum temperature, which varied from around -90 to -120°C across all experiments. Each sample from the different temperature steps are then cut into 1 mm thick slices and placed in 15mL of milliQ ultrapure water, and agitated overnight at 5°C to release the electrolytes into solution. Electrical conductivity was measured with a standard electrical conductivity meter (c_1), and then measured a second time (c_2) following autoclaving for 30 mins at 120°C to kill all living cells.

The ratio of the two conductivities, REL was calculated as follows:

$$REL = c_1/c_2 \tag{1}$$

Then a four-parameter logistic function was fit using nlme (Pinheiro *et al.*, 2023), with the self-starting function SSfpl (R Core Team, 2023). This function defines REL as a function of temperature (x), such as:

$$REL = a + (b - a)/(1 + e^{d*(x-c)}) \tag{2}$$

where c is the inflection point (i.e., LT_{50}), d is the slope at the inflection point and a and a+c the two asymptotes, the minimum and maximum REL at +5°C and at the lowest temperature, respectively. We fit one curve per species with individual as a random effect on the inflection point parameter c. We didn't include a random effect on the asymptotes because we lacked data to ensure the fit, and there seemed to be little variation of asymptotes across samples within a species.

Drought resistance database

Using available resources from botanical gardens and comparative growth trials, we collected stem samples from sun-exposed branches on 3-5 mature trees per species, aiming for straight segments with few/no side branches of around 1cm diameter. We used the flow centrifuge 'cavitron' technique (Cochard *et al.*, 2005; Burlett *et al.*, 2022) at the Caviplace lab (Bordeaux, France). This technique is vulnerable to the 'open-vessel' artefact, so we only measured species with short vessels based on vessel length estimates from the literature, and used a larger rotor size (29cm or 35cm) when necessary. Briefly, as the samples are spun in a custom centrifuge rotor, a water potential gradient is established through the sample connecting a large volume "upstream" reservoir to a smaller "downstream" one. A camera allows to monitor flow out of the upstream reservoir, which can be transformed to xylem specific conductivity by knowing the length and area of conductive tissue of the sample. The pressure in the xylem is related to the speed of the centrifuge, and as speed is gradually increased, the tension at the center of the sample induces embolism analogously to drought in the field, leading to a sigmoid-shaped decrease of hydraulic conductance. This relationship between percent loss of hydraulic conductance and water potential is known as a vulnerability curve. We fit to this relationship a sigmoid model (Pammenter & Vander Willigen, 1998) to extract the

inflection point (pressure inducing 50% loss of hydraulic conductance, P50), using either SAS (SAS Institute Inc., Cary, NC, USA) or the fitplc package in R (Duursma & Choat, 2017). In total, 23 new taxa were added to the database in the spring and early summer of 2021, 8 angiosperms and 15 conifers. Of these, 15 are included in the analyses of this paper, and 3 are already published (Larter *et al.*, 2024).

Bibliography

Burlett R, Parise C, Capdeville G, Cochard H, Lamarque LJ, King A , Delzon S. 2022. Measuring xylem hydraulic vulnerability for long-vessel species: an improved methodology with the flow centrifugation technique. *Annals of Forest Science*, **79**: 5.

Cochard H, Damour G, Bodet C, Tharwat I, Poirier M , Améglio T. 2005. Evaluation of a new centrifuge technique for rapid generation of xylem vulnerability curves. *Physiologia Plantarum*, **124**: 410–8.

Duursma R , Choat B. 2017. fitplc - an R package to fit hydraulic vulnerability curves. *Journal of Plant Hydraulics*, **4**: e002–e002.

Larter M, Akhmedov A, Payne C, Delzon S , Klein T. 2024. High and variable hydraulic resistance to embolism among three Asian juniper species: Hydraulic resistance to embolism in Asian junipers. *Journal of Plant Hydraulics*, **10**: 2–2. Number: 1.

Pammenter NW , Vander Willigen C. 1998. A mathematical and statistical analysis of the curves illustrating vulnerability of xylem to cavitation. *Tree Physiology*, **18**: 589–93.

Pinheiro J, Bates D , Team RC. 2023. nlme: Linear and Nonlinear Mixed Effects Models.

R Core Team. 2023. R: A Language and Environment for Statistical Computing.
